# Lineage Tracing of FOXL1+ Cells in the Tunica Muscularis Suggests Mutual Origin for Telocytes and Smooth Muscle Cells

**DOI:** 10.3390/life12020176

**Published:** 2022-01-26

**Authors:** Moriya Shushan, Michal Shoshkes-Carmel

**Affiliations:** Department of Developmental Biology and Cancer Research, The Hebrew University, Jerusalem 91120, Israel; moriya.shushan@mail.huji.ac.il

**Keywords:** FOXL1, SMCs, telocytes (TCs), interstitial Cajal cells (ICC)

## Abstract

We recently identified a FOXL1+ intestinal subepithelial network of telocytes (TCs) without which epithelial stem and progenitor cells cannot proliferate and support regeneration. In addition to FOXL1 lineage cell distribution along the intestinal epithelium, we also observed their presence within the muscle layers. Here, we characterized FOXL1+ lineage cells along the muscle layers of the duodenum in order to understand their progeny and relation to interstitial Cajal cells (ICCs), smooth muscle cells (SMCs) and the previously reported PDGFRa+ TCs. Using a FOXL1-Cre transgenic line in conjunction with genetic lineage labeling using the *Rosa26-mTmG* allele, in which Cre-marked cells produce a membrane-targeted version of green fluorescent protein (GFP), we found that within the muscle layers FOXL1 lineage GFP+ cells had two main progeny; (i) elongated multinucleated SMA+ SMCs, intermingled in parallel or perpendicular to muscle fibers. (ii) TCs displaying small cell body with multiple cell processes, expressing PDGFRa and CD34. These findings may suggest a mutual origin for TCs and SMCs.

## 1. Introduction

The intestine is an outstanding model with which to study cellular coordination and cooperation and how they are regulated to achieve functional tissue. The intestine’s main functions are to digest and absorb nutrients and water during propulsion of food along the gastrointestinal tract. In order to maintain its vital functions, a high degree of coordination between the different cell types and cellular components is required. The epithelium limits interactions between luminal residents, such as pathogens or the microbiota, and the host, including immune cells, and cooperates so that epithelial-cell-type composition does not change, and regeneration occurs. The enteric nervous system cooperates with smooth muscle cells (SMCs) to control motility, and intestinal vasculature is crucial for nutrient absorption and recruitment of immune cells.

Recently, in searching for the source of cells which provide the signals required for epithelial functional support, we identified mesenchymal cells expressing the transcription factor FOXL1 as an important source of Wnt proteins without which intestinal stem and progenitor cells cannot proliferate and support regeneration [1,2]. Using a FOXL1-Cre transgenic line in conjunction with genetic lineage labeling using the *Rosa26-mTmG* allele [3], in which Cre-marked cells produce a membrane targeted version of green fluorescent protein (GFP), we unexpectedly found that FOXL1-expressing cells belong to a novel type of mesenchymal cells called telocytes (TCs) that form a continuous comprehensive three-dimensional network of contact with the entire intestinal epithelium, from the crypt base to the tip of the villi. In support of our work, recent studies from different research groups used diverse markers to label TCs and demonstrated their importance as a critical stem cell niche component that supports intestinal crypts [4,5,6].

TCs are characterized by cytoplasmic processes that are separated from the epithelium by sub-micrometer distances. Intestinal subepithelial TCs express the surface membrane platelet-derived growth factor receptor α (PDGFRα), while the transcription factor FOXL1 is expressed in their nuclei. TCs, primarily described using electron microscopy (EM), were observed by Popescu and Faussone-Pellegrini to be peculiar cells in the intestinal muscle layers which are structurally similar but distinct from the interstitial Cajal cells (ICCs) [7,8,9,10,11,12,13,14,15]. PDGFRa+ cells, located in the muscle layers together with SMCs and ICCs, were previously reported to form an electrical syncytium that coordinates motility [16].

Here, we used our transgenic mouse line FOXL1-Cre: Rosa-mTmG, in which FOXL1 Cre drives the expression of a membrane-bound GFP and performed whole mounting as well as section staining for c-kit to label ICCs, SMA for SMCs and PDGFRa or CD34 for TCs, to characterize FOXL1 lineage cells in the muscle layers and understand their relations and interactions to the above-mentioned cell types. We found that, in the muscle layers, FOXL1 lineage GFP+ cells had two main cellular characteristics: (i) elongated multinucleated SMA+ muscle cells, intermingled in parallel or perpendicular to muscle fibers. (ii) A TC-like structure displaying a flat cell body with multiple cell processes, expressing PDGFRa and CD34. These findings may suggest a mutual origin between SMCs and TCs linked by FOXL1 lineages.

## 2. Materials and Methods

### 2.1. FOXL1Cre: Rosa-mTmG Mice

Mice were obtained by crossing a FOXL1-Cre transgenic line in conjunction with genetic lineage labeling using the *Rosa26-mTmG* allele [3] as described previously [1,2].

### 2.2. Immunofluorescence Staining

Mouse intestines were rinsed in PBS and fixed with 4% paraformaldehyde overnight at 4 °C, washed in PBS, and immersed in 30% sucrose overnight at 4 °C, then embedded in OCT in order to create cryoblocks. Blocks were serially sectioned at 10 µm.

The following antibodies were used: goat anti-PDGFRa (R&D cat:AB1066; 1:200), chicken anti-GFP (Novos Bio cat no: NB100–1614; 1:500), goat anti-GFP (Abcam cat no: AB6673; 1:200), rat anti c-KIT (CD117; Invitrogen Thermo Fisher cat no: 14-1172-82; 1:500).

### 2.3. Clearing Mouse Intestine Using X-Clarity^TM^

Mice were sacrificed and their duodenums dissected and fixed at 4%PFA for 24 h while shaking at 4 °C. Following washes with PBS for additional 24 h at 4 °C, tissue was incubated in hydrogel solution (according to manufacturer’s protocol) for 24 h at 4 °C. Following a further 3 h incubation at 37 °C, the hydrogel-embedded tissue was placed in an X-CLARITY ETC chamber (LOGOS Biosystems) for electrophoretic tissue clearing for 7 h. The cleared duodenum was immunostained with goat anti-PDGFRa (1:100), chicken anti-GFP (1:100), rat anti-c-KIT (1:200), goat anti-GFP (1:500), or rabbit anti-SMA (Abcam 1:600). Primary and secondary antibodies were incubated for 48 h at 4 °C. The stained intestine was placed en bloc on an image slide using a 0.5 mm depth adhesive silicone isolator, mounted in X-CLARITY mounting solution and imaged using confocal scanning. Z-stack projections were compiled.

## 3. Results

Here, we used a transgenic reporter mouse in which FOXL1 Cre drives the expression of a membrane-tag GFP in order to understand the identity of FOXL1 lineage cells and their relations to ICCs, SMCs and PDGFRa+ CD34+ TCs.

### 3.1. The Distribution of FOXL1 Lineage GFP+ Cells in the Tunica Muscularis

**Figure 1:**
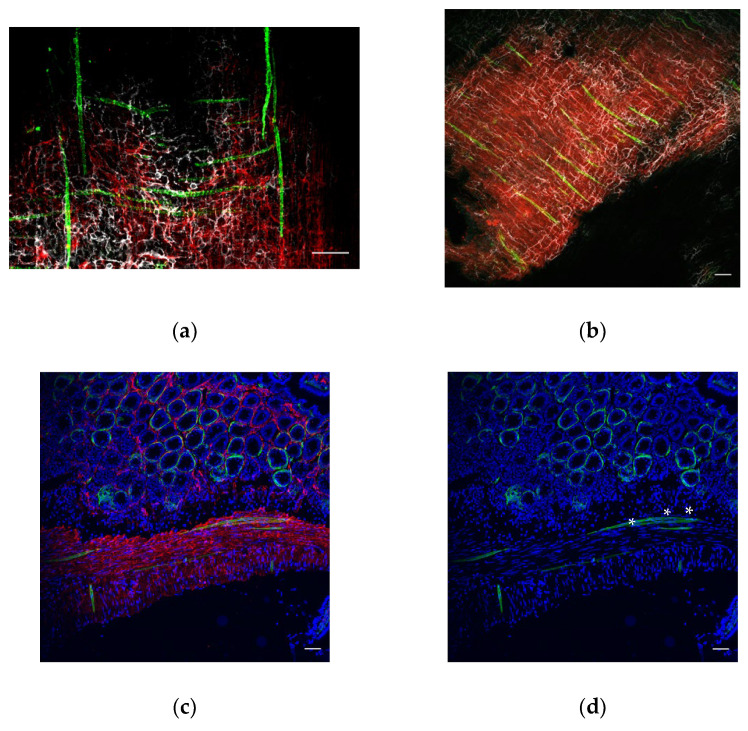
FOXL1 lineage GFP+ cells distributed throughout the muscle layers belong to muscle cells: representative images of whole cleared mount (**a**,**b**) or sections (**c**,**d**) of the mouse duodenum of the FOXL1Cre: Rosa-mTmG mouse line stained for (**a**,**b**) GFP (green), c-kit (red) and PDGFRa (white); (**a**) representative image of mouse duodenal muscle layer showing the distribution of GFP+ cells in parallel or perpendicular to longitudinal smooth muscle. (**b**) Representative image of the mouse duodenal muscle layer showing the distribution of GFP+ cells in parallel to longitudinal muscle. (**c**,**d**) GFP (green), SMA (red). Note the co-expression of GFP and SMA in muscle multinucleated cells (**d** asterisks point on nuclei). Scale bar 50 μm.

The intestinal tunica muscularis contains two layers of smooth muscles. The inner layer consists of circular muscles, whereas the outer muscle fibers are oriented longitudinally. In order to give a broad view on the distribution of FOXL1 GFP+ lineage cells throughout the tunica muscularis, we used our transgenic mouse line FOXL1 Cre: Rosa-mTmG, cleared a whole duodenum (Figure 1a,b) and stained for GFP (green) to follow FOXL1+ lineage cells, using a c-kit to label ICCs (red) and PDGFRa (gray) to label the previously described TCs. Interestingly, most of the FOXL1 lineage GFP+ cells at the muscle layers were negative for PDGFRa expression. FOXL1 lineage cells showed a striated elongated structure and were distributed in parallel or perpendicular to longitudinal muscle fibers negative for c-kit or PDGFRa expression. Staining for SMA to label SMCs (Figure 1c red) confirmed that FOXl1 lineage cells distributed in the muscle layers belong to a fraction of multinucleated muscle cells (in Figure 1d asterisks point to nuclei).

### 3.2. FOXL1 Lineage GFP+ Cells in Relation to Interstital Cajal Cells (ICCs)

**Figure 2:**
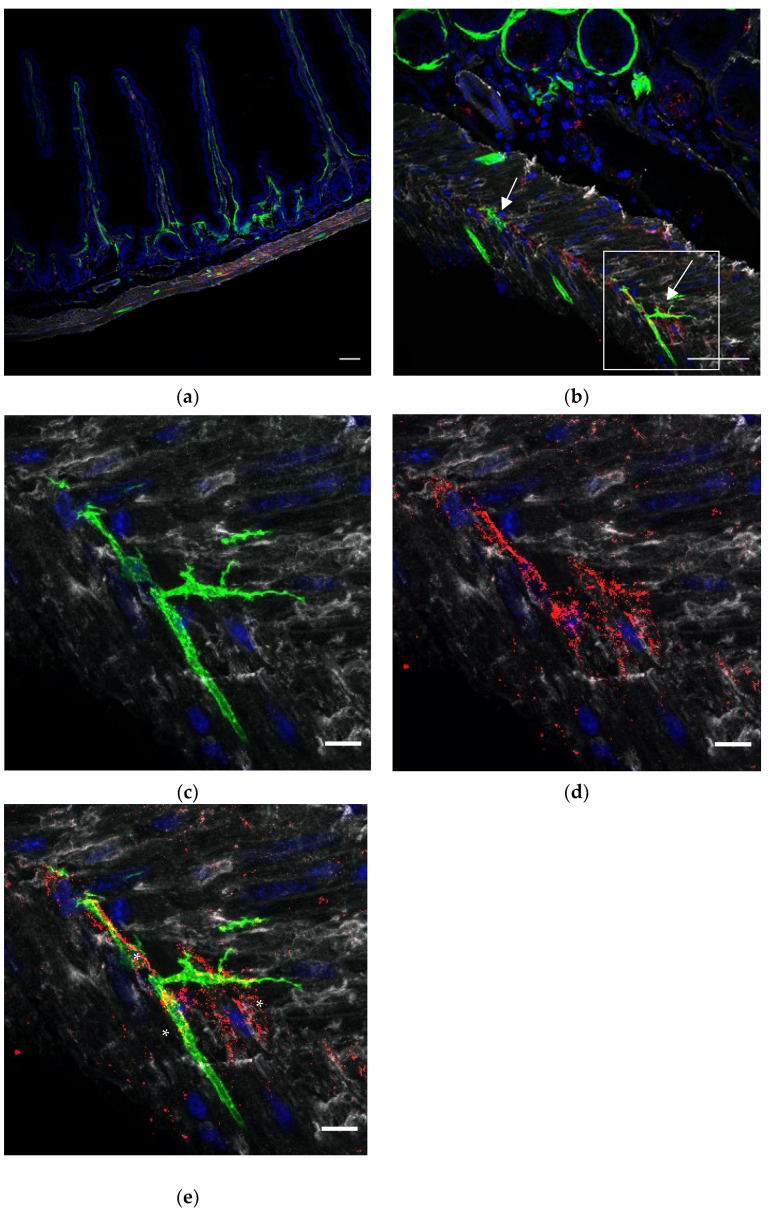
A small fraction of FOXL1 lineage GFP+ cells which show TC characteristics are in close association to ICCs and SMCs: representative images of duodenal sections of the FOXL1Cre: Rosa-mTmG mouse line stained with GFP (green), smooth muscle actin (SMA) (white) and c-kit (red). (**a**) Longitudinal section showing the subepithelial and muscle distribution of GFP+ cells. (**b**) The duodenal muscle layers. Please note the distribution of GFP+ cells which show TC characteristics (**b** arrows) in addition to the elongated GFP+ muscle cells. (**c**–**e**) High magnification of the region depicted in (**b**). The overlapping staining (**e**, asterisks) represents sites of cell–cell contact, suggesting interactions between GFP+ and ICCs. Scale bar 50 μm (**a**,**b**), 10 μm (**c**–**e**).

ICCs form networks throughout the intestinal wall, close to nerve endings and SMCs to regulate motility. In order to appreciate the relations between GFP+ FOXL1 lineage cells to ICCs or SMCs, we used cryosections of our transgenic line FOXL1 Cre: Rosa-mTmG and co-stained for GFP, c-kit and smooth muscle actin (SMA) to label FOXL1 lineage cells, ICCs and SMCs, respectively. In addition to GFP+ SMA+ muscle cells, we noticed that a small fraction of the GFP+ cells showed typical TC-like characteristics (Figure 2b arrows), cells with small cell body and multicellular processes. Close interactions between GFP+ cells ICCs and SMCs were observed (Figure 2c–e). The overlapping staining in yellow (Figure 2e asterisks) represents sites of cell–cell contact suggesting potential communication between GFP+ cells ICCs and SMCs.

### 3.3. FOXL1 Lineage GFP+ in Relation to PDGFRa+ or CD34+ TCs

**Figure 3:**
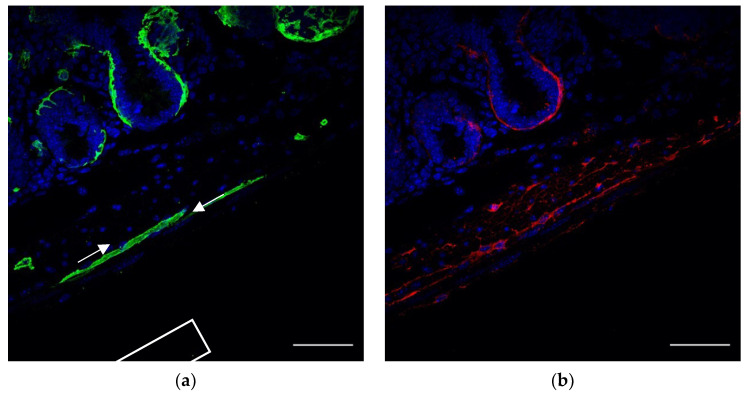

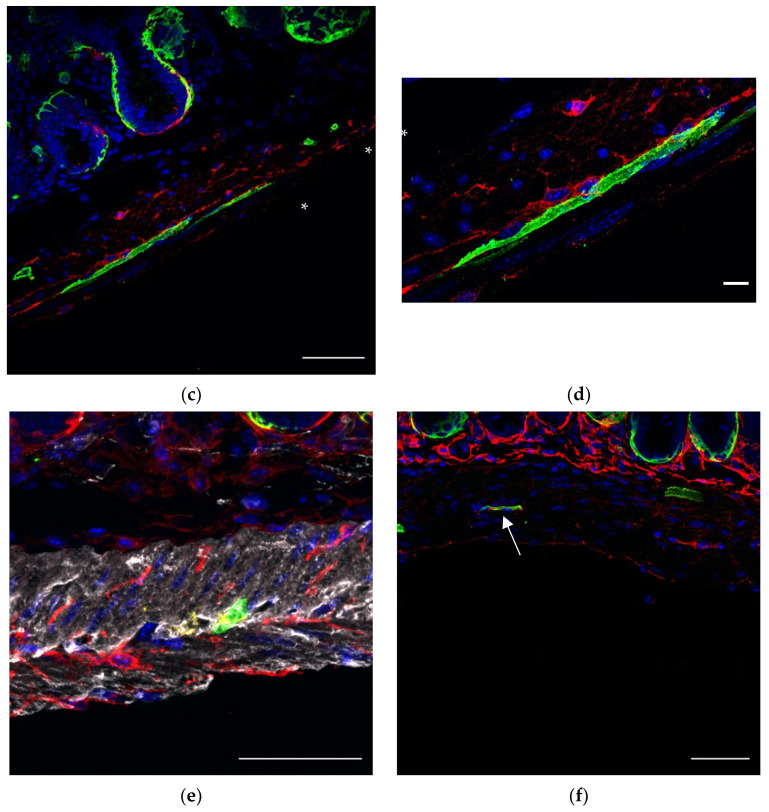
A small fraction of FOXL1 lineage GFP+ cells which showed TC characteristics express PDGFRa or CD34: representative images of duodenal sections of the FOXL1Cre: Rosa-mTmG mouse line co-stained for (**a**) GFP (green) (**b**) PDGFRa (red) (**c**) the merged image. Note that, whereas the subepithelial network of TCs consist mostly of double positive GFP, PDGFRa expression (Figure 3c arrows), the GFP+ elongated muscle cells are PDGFRa-negative (Figure 3c asterisk). (**d**) High magnification of the region depicted in (**c**). Note the interaction between GFP+ muscle and PDGFRa+ cells represented by overlapping staining at sites of cell–cell contact. (**e–f**) Representative images of duodenal sections of the FOXL1Cre: Rosa-mTmG mouse line co-stained for GFP (green) and (**e**) PDGFRa (red) or (**f**) CD34 (red). Note the co-expression of GFP with PDGFRa or CD34. Scale bar 50 μm (**a**–**c**,**e**,**f**), 10 μm (**d**).

In order to confirm whether FOXL1 lineage GFP+ cells which exhibit typical TC-like characteristics indeed belong to TCs and to characterize the relations between GFP+ cells and TCs, we co-stained sections of our transgenic lines for PDGFRa or CD34 (Figure 3). Whereas most of the subepithelial network of TCs consist of double positive GFP-, PDGFRa-expressing cells (Figure 3c arrows), most of the GFP+ cells along the muscle layer showed SMC characteristics negative for PDGFRa (Figure 3c asterisk). Notably, close association between GFP+ and PDGFRa+ cells was observed, suggesting potential communication between the cells. As we mentioned earlier, a small fraction of GFP+ cells along the muscle layers showed the typical TC-like structure of cells with multicellular processes; in order to test whether this fraction of cells belongs to TCs, we stained for PDGFRa or CD34. Indeed, GFP+ cells were positive for PDGFRa (Figure 3e red) and CD34 (Figure 3f red), representing a small fraction of the overall PDGFRa+ or CD34+ cells.

## 4. Discussion

TCs have been described primarily by EM, defined by their structure as mesenchymal cells with a small cell body and long processes, called telopodes. These telopodes may reach a length of 100 microns and consist of dilated segments containing mitochondria, rER and caveolae [11,12,13,14,15]. The attempts to try and find specific markers which label TCs by performing immunofluorescent staining using markers such as SMA, CD34 or PDGFRa proved challenging as TCs are heterogeneous in their gene expression profile according to the organ, their tissue location, condition, etc. [2,3,7,8,9,10,11,12,13,14].

In the gut, TCs were considered as CD34-positive c-kit-negative, PDGFRa-positive c-kit-negative or double PDGFRa CD34-positive c-kit-negative, although PDGFRa and CD34 also label endothelial cells, emphasizing the complexity in trying to find one specific marker to label TCs [9].

We recently identified a subepithelial network of FOXL1+ cells along the entire intestinal crypt–villus axis [1,2]. Using a transgenic reporter mouse line in which FOXL1 Cre drives the expression of a membrane-tag GFP, which allows us to visualize the structure of FOXL1+ cells, and based on EM and immune-staining for PDGFRa, we found that the subepithelial FOXL1+ cells are mesenchymal TCs. Most of the subepithelial network of TCs express both PDGFRa and FOXL1, although a portion of the cells were PDGFRa+ only, distinct from the FOXL1+ lineage cells and a portion of the FOXL1+ lineage cells were PDGFRa-negative.

In addition to FOXL1+ TCs’ subepithelial distribution, we also observed GFP+ cells along the muscle layers. We characterized FOXL1 lineage GFP+ cells along the muscle layers of the duodenum in order to understand their identity and relation to ICCs and the previously described PDGFRa+ or CD34+ TCs.

We found that GFP-labeled FOXL1 lineage cells located along the muscle layers have two main cellular features: (i) a smooth striated elongated fiber-like structure SMA-expressing muscle cell and (ii) a typical TC structure of a small cell body with multiple cytoplasmic processes expressing PDGFRa or CD34. Cells were oriented in parallel and perpendicular to muscle fibers, ICCs or PDGFRa+ cells and showed close association with each cell type. Notably, along the muscle layers the GFP+ FOXL1+ lineage cells represented a small fraction from the SMA+ SMCs or the PDGFRa+/CD34+ TCs.

Interestingly, similar to our findings using a FOXL1 cre driven reporter mouse line, PDGFRa+ lineage tracing experiments revealed similar cell progeny in the intestine with the highest level of recombination of PDGFRa+ becoming GFP in SMCs [17]. A possible explanation for these cells is that FOXL1+ PDGFRa+ cells share a mutual cell origin of a progenitor population with differentiation capabilities to SMCs. However, at the moment we cannot exclude possible miss-expression of the transgene.

A detailed lineage-tracing of FOXL1+ cells and their descendants over time together with transcriptional analysis at a single cell level in combination with spatial tissue characterization would clarify the identity of FOXL1 progeny. The FOXL1 progeny of SMCs and TCs may suggest mutual origin for the two cell types.

The close association between GFP+ SMCs and PDGFRa+ cells and ICCs suggest potential interactions between the cells and a possible role in regulating intestinal motility.

## Data Availability

Not applicable.
